# The varied Q creatinine in multi ethnics population and impact of adopting three different estimated glomerular filtration rates based on creatinine in adult populations: a call for performance validation

**DOI:** 10.3389/fmed.2025.1467503

**Published:** 2025-03-24

**Authors:** Ferdy Royland Marpaung, Santi Wulan Purnami, Shofi Andari, Ali Rohman, Reny I’tishom, Hari Basuki Notobroto, Jusak Nugraha, Risky Vitria Prasetyo, Djoko Santoso, Etienne Cavalier, Aryati Aryati

**Affiliations:** ^1^Doctoral Program of Medical Science, Faculty of Medicine, Universitas Airlangga, Surabaya, Indonesia; ^2^Department of Statistics, Institut Teknologi Sepuluh Nopember, Surabaya, Indonesia; ^3^Department of Chemistry, Faculty of Science and Technology, Universitas Airlangga, Surabaya, Indonesia; ^4^Department of Biomedical Science, Universitas Airlangga, Surabaya, Indonesia; ^5^Faculty of Public Health, Universitas Airlangga, Surabaya, Indonesia; ^6^Department of Clinical Pathology, Faculty of Medicine, Universitas Airlangga, Surabaya, Indonesia; ^7^Department of Child Health, Faculty of Medicine, Universitas Airlangga, Surabaya, Indonesia; ^8^Department of Internal Medicine, Faculty of Medicine, Universitas Airlangga, Surabaya, Indonesia; ^9^Department of Clinical Chemistry, University of Liège, CIRM, CHU Sart Tilman, Liège, Belgium

**Keywords:** creatinine, Q creatinine, EGFR, kidney function, non-communicable disease

## Abstract

**Background:**

The determination of kidney function is commonly done by estimating the glomerular filtration rate (eGFR) using serum creatinine levels. Various eGFR formulas, including the recently developed European Kidney Function Consortium (EKFC) and the Chronic Kidney Disease Epidemiology Collaboration(CKD-EPI), have been adopted and are commonly utilized in clinical settings. Nevertheless, the extent of acceptance among these formulations in the multi ethnics populace is still undetermined. Thus, this study aimed to evaluate the performance of these formulations across different glomerular filtration rate categories in the adult population.

**Methods:**

The research involved a total of 9,557 individuals (median age of 40 years and 85% being male) who underwent routine medical examinations. Enzymatic or modified Jaffe techniques were employed to measure serum creatinine levels. The CKD-EPI2009 eGFR was employed as corresponding GFR in the comparisons. The Bland–Altman method was used to determine the average discrepancies and 95% confidence intervals of eGFR between each formula. Ultimately, in order to compare the equations, Lin’s correlation coefficients were calculated for various eGFR categories.

**Results:**

The median creatinine level in the different island population showed variability. The CKD-EPI 2009 as well as different equations showed categorical agreement within the range of 91.42 to 92.77%. The correlations between CKD-EPI2009 and CKD-EPI 2021 and EKFC were 0.998 and 0.79, respectively (*p* < 0.001).

**Conclusion:**

A substantial variation in creatinine and eGFR assessment were observed among different eGFR analysis for the adult population. Prospective study in various clinical contexts using measured GFR is essential to validate eGFR.

## Introduction

It is recommended to estimate the glomerular filtration rate (GFR) and albuminuria for assessing renal function and detection of chronic kidney disease (CKD) in adults (1). Furthermore, determining GFR is crucial for monitoring therapy achievement, disease progression, drug dosage, and drug toxicity (2, 3). The substances widely utilized to evaluate GFR are endogenous compounds, particularly creatinine (4). Numerous formulas known to be aimed at estimating GFR (eGFR) by measuring creatinine are used to evaluate renal function. The Chronic Kidney Disease Epidemiology Collaboration (CKD-EPI) equation 2009 and 2021 (5, 6), and the recently established European Kidney Function Consortium (EKFC) (7) formula are commonly utilized in clinical settings (8).

Several studies have been conducted in Asian countries using their specific demographics to validate eGFR (%^9^–^12^). Their respective studies are important since most formulas are generated from European and American-based populations. It was found that the equations exhibited bias in their respective populations. To address this, ethnic coefficients were created to adapt the formula for Modification Diet for Renal Disease (MDRD), and later the CKD-EPI. Asian people exhibit a distinct normal range for creatinine compared to Caucasian populations, potentially because to variations in muscle mass, food, genetics, and other factors (9, 13).

Indonesia, an archipelagic country with five main islands, is home to diverse ethnic characteristics that may have the potential to impact serum creatinine levels, which in turn will influence the use of the eGFR formula. While previous studies have compared eGFR equations in other Asian populations, no prior research has systematically evaluated the performance of CKD-EPI 2009, CKD-EPI 2021, and EKFC in the Indonesian population. Given Indonesia’s unique demographic and ethnic diversity, assessing the applicability of these equations is crucial. This study provides the first large-scale comparison of these formulas in an Indonesian cohort, offering new insights into their performance and potential limitations in this specific population.

## Methods

### Participants

The individuals of regular medical screening at the Pramita Clinic Laboratory (Indonesia) were consecutively included in August 2023 who met the following criteria: age ≥ 17 years and presence of creatinine serum findings. There were no exclusion criteria, except for patients referred from the Nephrology Clinic or sent by the nephrologist. Pramita Clinic Laboratory is a private laboratory specialist health-screening facility that delivers standard medical check-ups for 1,000,000–1,500,000 people per year in 38 branch laboratories in Indonesia.

**Figure 1:**
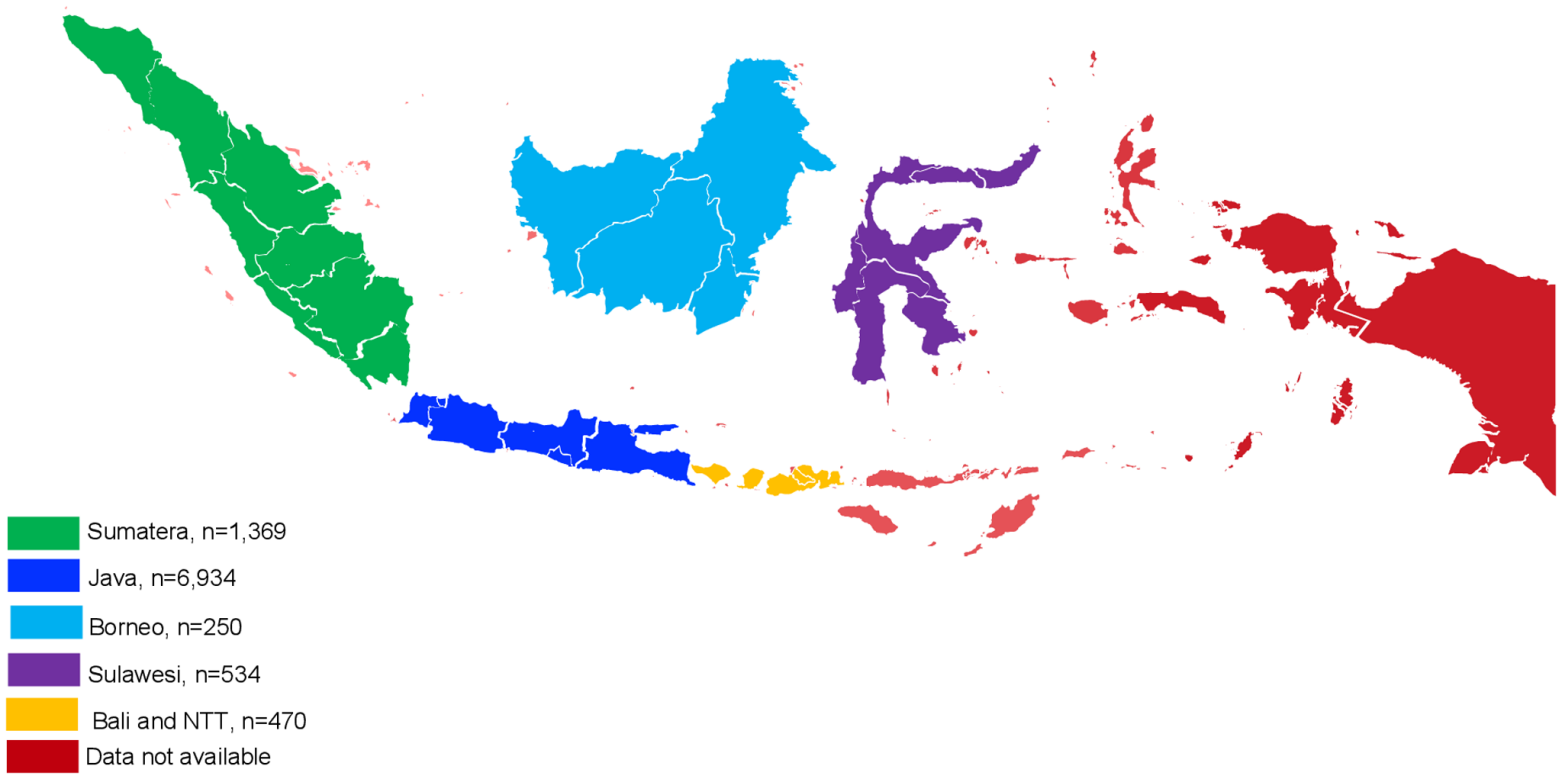
Participants distribution in Indonesia region.

Indonesia is an archipelagic country with five major islands (Sumatera, Java, Borneo, Sulawesi, and Bali/NTT) (Figure 1), each with distinct ethnic compositions and lifestyle factors that could influence creatinine metabolism. The division into these five regions follows Indonesia’s administrative and demographic classifications, which are commonly used in epidemiological and public health studies. This regional approach allows us to capture potential variations in creatinine levels and eGFR estimations across the country. There were 9,557 patients with serum creatinine values (median age: 40 years old, and 85% male). Most of the participants were male, as the data was collected from employees who underwent yearly medical screenings through cooperation with Pramita laboratories. A significant proportion of these companies exhibit a larger number of male staff members. We performed a retrospective study of the laboratory findings utilizing the three eGFR equations (CKD-EPI 2009, CKD-EPI 2021, and EKFC). This study This study has been approved by the Dr. Soetomo Academic Hospital/Faculty of Medicine Universitas Airlangga Institutional Review Board (No: 0938/III/2024).

### GFR equation and classification

Creatinine examination was performed using the enzymatic on an automated chemistry analyzer (Architect Abbott, USA), except for Borneo island (303 participants) the creatinine was testing using Kinetic Jaffe (Architect Abbott, USA). All examinations were traceable using an IDMS calibrator (Calibrator list number Abbot, USA: 08P65/6 K30 and 08P60/1E65 for enzymatic and kinetic Jaffe, respectively). Two concentration levels of Lyphochek-assayed clinical chemistry quality control materials (manufactured by Bio-Rad, Hercules, CA, USA) were subjected to internal quality control testing in the creatinine assay, conducted twice daily. During the study period, the serum creatinine assay had a coefficient of variation (CV) within-laboratory precision of 1.19% for QC level 1 and 0.67% for QC level 2. The laboratory participated in the Indonesian quality assurance for clinical laboratories external proficiency testing program (BBLK) in 2023, and the results were all satisfactory (Z-scores between 0.14 and 1.09%).

The calculated GFR of EKFC, CKD-EPI 2021 and CKD-EPI 2009 were explained in the previous study (6, 7, 14). We did not incorporate any factors into the equation of CKD-EPI. For the EKFC, we calculated the median creatinine for men and women aged 17–40 and > 40 years from the 9,557 available data points. The study revealed that the median creatinine levels for individuals aged 17–40 years were 0.98 mg/dL for male and 0.67 mg/dL for female. In contrast, for individuals aged over 40 years, the median creatinine levels were 1.0 mg/dL for male and 0.70 mg/dL for female. Based on the KDIGO 2012 guidelines, the glomerular filtration rates (GFRs) are categorized as follows: G1 for GFRs of 90 or above, G2 for GFRs between 60 and 89, G3a for GFRs between 45 and 59, G3b for GFRs between 30 and 44, G4 for GFRs between 15 and 29, and G5 for GFRs of 15 or lower, all measured in mL/min/1.73 m2. The occurrence of reduced glomerular filtration rate (eGFR <60 mL/min/1.73 m2) was assessed and compared among the three equations derived from blood creatinine levels (15).

### Statistical analysis

The eGFR CKD-EPI 2009 was used as the reference GFR for comparisons. The Bland–Altman plots were used to establish the mean differences and 95% limits of agreement of eGFRs between each equation and the CKD-EPI 2009 equation. In order to contrast the equations, Lin’s concordance correlation coefficients (r) were calculated for various GFR groups using the provided classification: r coefficients of 0.65 or less were regarded as poor correlations, 0.65–0.8 were considered moderate correlations, and 0.8–0.9 were considered substantial correlations, and r levels of >0.90 being almost perfect correlations (16). When eGFR CKD-EPI and other equations fell into the same GFR category, the categorical agreement rates were determined. The level of agreement between categories was assessed by calculating a weighted kappa value. The agreement between diagnoses was examined using a kappa value, with GFR cut-off of 60 and 45 mL/min/1.73m^2^. The following kappa coefficients were assigned: bad (<0.20), fair (0.21–0.40), moderate (0.41–0.60), good (0.61–0.80), and very good (>0.81) (17). Reduced GFR is defined as an eGFR of <60 mL/min/1.73 m^2^. Data were analyzed using SPSS 27 (IBM, USA), and *p* values ≤0.05 were regarded to be statistically significant.

## Result

### Subject characteristics

The majority of the study population was from 38 branch laboratories distributed in western Indonesia (75%), most commonly from Java Island. Table 1 displays the baseline participant demographics and estimated eGFR categorized by age group. The magnitude of the discrepancy varied according to the formula and the age range. The CKD-EPI 2021 equations yielded higher mean eGFRs in individuals compared to other equations used in this research. Nevertheless, the CKD-EPI 2009 equation yielded the lowest eGFR in individuals above the age of 60 when compared to other equations.

**Table 1:** Participant demography and group of estimated glomerular filtration rates classification.

Variable	17–29 yr	30–39 yr	40–49 yr	50–59 yr	>60 yr	Total	*P*-value
	*N* = 1,698	*N* = 3,021	*N* = 3,323	*N* = 1,417	*N* = 98	*N* = 9,557	
Male %	79.92	87.98	86.76	83.70	43.88	85.04	
Age (Median)	26	35	44	52	67	40	
Body mass index (kg/m^2^) Mean ± SD	24.6 ± 5.01	25.49 ± 4.61	25.62 ± 4.08	25.92 ± 3.94	24.54 ± 4.29	25.43 ± 4.42	
Serum-Cr (mg/dL)							
Male (median)	0.97	0.97	0.97	1.00	1.01	0.98	0.000
Female (Median)	0.66	0.68	0.69	0.72	0.71	0.69	0.000
eGFR_CKD-EPI 2009_ (mL/min/1.73 m^2^) mean ± SD	109.43 ± 14.48	101.44 ± 14.23	94.43 ± 14.63	85.52 ± 15.58	78.29 ± 18.65	97.82 ± 16.53	0.000
eGFR_CKD-EPICreat 2021_ (mL/min/1.73 m^2^) mean ± SD	111.76 ± 13.95	104.49 ± 13.95	97.96 ± 14.49	89.38 ± 15.73	81.97 ± 18.61	101.03 ± 16.14	0.000
EKFC (mL/min/1.73 m^2^) mean ± SD	104.04 ± 9.87	103.71 ± 10.8	101.74 ± 14.39	91.91 ± 16.41	82.81 ± 17.94	82.81 ± 17.94	0.000

Data presented as mean ± standard deviation except male (%), creatinine (median), age (median). yr, year; Cr, creatinine; eGFR, estimated glomerular filtration rate; CKD-EPI, Chronic Kidney Disease Epidemiology Collaboration; EKFC, European Kidney Function Consortium.

### Qcr and BMI in different population

The median creatinine level test comparing genders in each region reveals a substantial disparity in creatinine values between men and women. When compared to other islands, the Bali/NTT population had the highest serum creatinine than other regions, both in men (1.02 mg/dL) and women (0.75 mg/dL), while Borneo had the lowest median creatinine serum, both in men (0.90 mg/dL) and women (0.60 mg/dL). These findings are summarized in Table 2. However, in the Balinese/NTT population, there was no notable disparity in the Body Mass Index (BMI) between males and females. There were variations in the creatinine values observed in different male groups, except in specific regions such as Sumatera compared to Sulawesi, Sumatera compared to Bali/NTT, and Sulawesi compared to Bali/NTT. Furthermore, there was no significant difference in the creatinine level among the female population of Sumatera compared to Bali and Borneo compared to Sulawesi (Table 3).

**Table 2:** Qcr and BMI in various population.

Population *N* = 9,557	Qcr (mg/dL)				BMI			
	Male median (IQR)	Female median (IQR)	Difference	*P* value	Male Mean ± SD	Female Mean ± SD	Mean difference (95% CI)	*P* value
Sumatera (*n* = 1,104)	*N* = 958 (0.18)	*N* = 146 0.7 (0.2)	0.1090	0.000	24.93 ± 4.21	26.03 ± 5.12	−0.675 (−0.97793–(−0.37308))	0.000
Java (*n* = 7,201)	*N* = 6,232 0.97 (0.19)	*N* = 969 0.69 (0.15)	−0.0055	0.000	25.31 ± 4.38	25.98 ± 4.97	−1.101 (−1.85942 – (−0.34380))	0.004
Borneo (*n* = 248)	*N* = 173 0.90 (0.2)	*N* = 75 0.60 (0.1)	0.0700	0.001	25.74 ± 4.22	24.47 ± 4.04	1.278 (0.14136–2.41493)	0.028
Sulawesi (*n* = 534)	*N* = 386 1.01 (0.21)	*N* = 148 0.67 (0.16)	−0.0723	0.002	25.99 ± 4.21	27.14 ± 4.95	−1.154 (−1.99497 – (−0.31327))	0.007
Bali/NTT (*n* = 470)	*N* = 378 1.02 (0.19)	*N* = 92 0.75 (0.13)	−0.1845	0.000	25.58 ± 3.82	26.36 ± 3.82	−0.781 (−1.65606–0.09230)	0.079

Q, median; Cr, creatinine; IQR, interquartile range; SD, standard deviation; CI, confidence interval.

**Table 3:** Median creatinine difference in male and female across the island.

Region	Male		Female	
	Difference median	*P*-Value	Difference median	*P*-Value
Sumatera vs. Java	0.04	0.000	0.04	0.002
Sumatera vs. Borneo	0.11	0.000	0.14	0.000
Sumatera vs. Sulawesi	−0.01	0.781	0.05	0.001
Sumatera vs. Bali/NTT	−0.01	0.193	−0.03	0.236
Java vs. Borneo	0.07	0.000	0.10	0.000
Java vs. Sulawesi	−0.05	0.000	0.01	0.047
Java vs. Bali/NTT	−0.05	0.000	−0.07	0.000
Borneo vs. Sulawesi	−0.12	0.000	−0.09	0.081
Borneo vs. Bali/NTT	−0.12	0.000	−0.17	0.000
Sulawesi vs. Bali/NTT	0.00	0.413	−0.08	0.000

### GFR frequency and eGFR

The total prevalence of CKD with an eGFR below 60 mL/min/1.73 m^2^, as determined by three different equations, ranged from 0.89 to 1.52%. The CKD-EPI 2009 equation had the greatest prevalence rate, while the EKFC equation had the lowest. Table 4 shows the prevalence of participants in each GFR group. There was a considerable difference in the distribution of each GFR type among the equations, specifically for G1 and G2. The proportions of G1 were 71.62% to 83.22 among three formulas. On the other hand, the prevalence of G2 ranged from 15.88 to 26.79%. According to the EKFC equation, the majority of the study group (> 80%) was in the G1 category.

**Table 4:** GFR distribution classification by three eGFR formula.

(mL/min/1.73m^2^)		CKD-EPI 2009	EKFC	CKD-EPI 2021
G1	>90	6,845 (71.62)	7,953 (83.22)	7,454 (78)
G2	60–89	2,560 (26.79)	1,518 (15.88)	1,984 (20.76)
G3a	45–59	110 (1.15)	60 (0.63)	83 (0.87)
G3b	30–44	27 (0.28)	17 (0.18)	24 (0.25)
G4	15–29	9 (0.09)	4 (0.04)	7 (0.07)
G5	1–14	6 (0.06)	5 (0.05)	5 (0.05)
Total		9,557 (100)	9,557 (100)	9,557 (100)

### Agreement CKD EPI 2009 and other equations in GFR classification

The level of agreement among the CKD-EPI 2009 and other equations ranged from 91.42 to 93.77% (Table 5). EKFC experienced an upward reclassification from G2 to G1. Weighted kappa scores was good in EKFC equations, and the overall concordance rate was more than 90%. The agreement between CKD-EPI 2009 and other were very good (CKD-EPI 2021) and good (EKFC) for cut-off value of 60 and 45 mL/min/1.73m^2^.

**Table 5:** Agreement between CKD-EPI 2009 and CKD-EPI 2021, EKFC formula.

	GFR category	CKD-EPI 2009							Categorical agreement		Diagnostic agreement at specific eGFR cutoffs	
		G1	G2	G3a	G3b	G4	G5	Total	%	Weighted kappa (95% CI)	Kappa at 60 mL/min/1.73 m2 (95% CI)	Kappa at 45 mL/min/1.73 m2 (95% CI)
CKD-EPI 2021	G1	6,662	641	0	0	0	0	7,303	92.76	0,836 (0,823 - 0,847)	0,647 (0,553 - 0,741)	0,702 (0,554 - 0,850)
	G2	0	2,084	38	0	0	0	2,122				
	G3a	0	0	85	9	0	0	94				
	G3b	0	0	0	21	3	0	24				
	G4	0	0	0	0	8	0	8				
	G5	0	0	0	0	0	6	6				
	Total	6,662	2,725	123	30	11	6	9,557				
EKFC	G1	6,561	1,244	0	0	0	0	7,805	84.90	0,635 (0,618 - 0,653)	0,414 (0,315 - 0,512)	0,437 (0,266 - 0,608)
	G2	101	1,481	72	0	0	0	1,654				
	G3a	0	0	51	17	0	0	68				
	G3b	0	0	0	13	8	0	21				
	G4	0	0	0	0	3	1	4				
	G5	0	0	0	0	0	5	5				
	Total	6,662	2,725	123	30	11	6	9,557				

### CKD-EPI and other eGFR equations correlation and concordance

**Figure 2:**
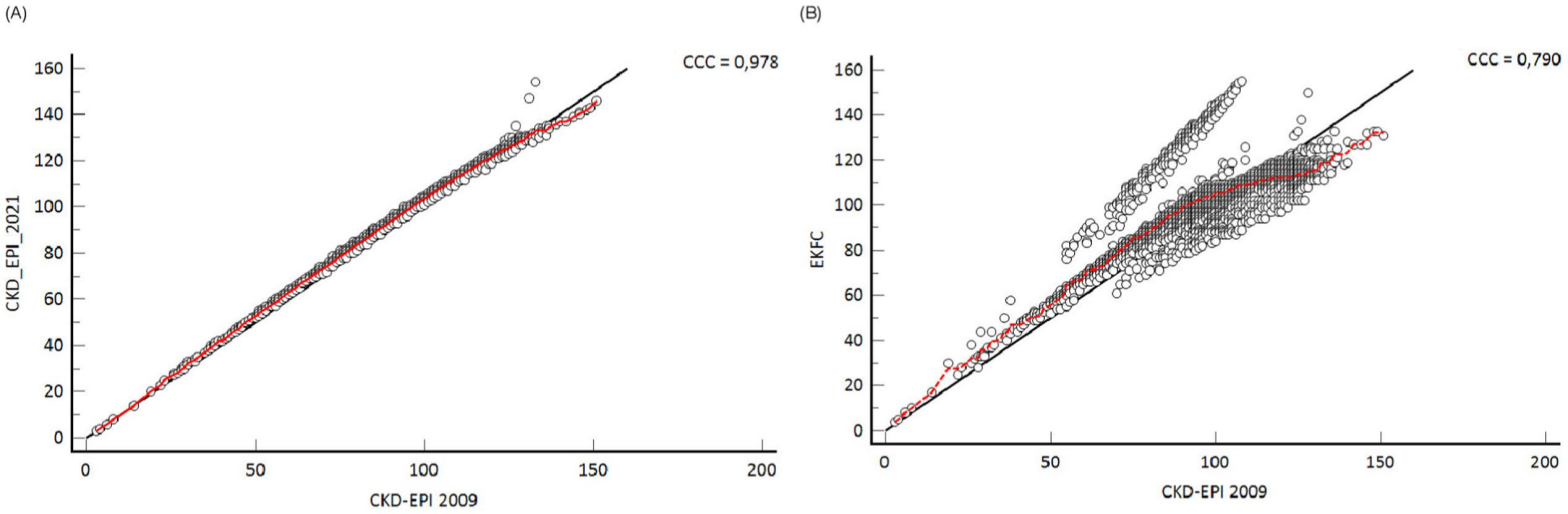
Correlation between **(A)** CKD-EPI 2009 and CKD-EPI 2021, and **(B)** CKD-EPI 2009 and EKFC.

**Figure 3:**
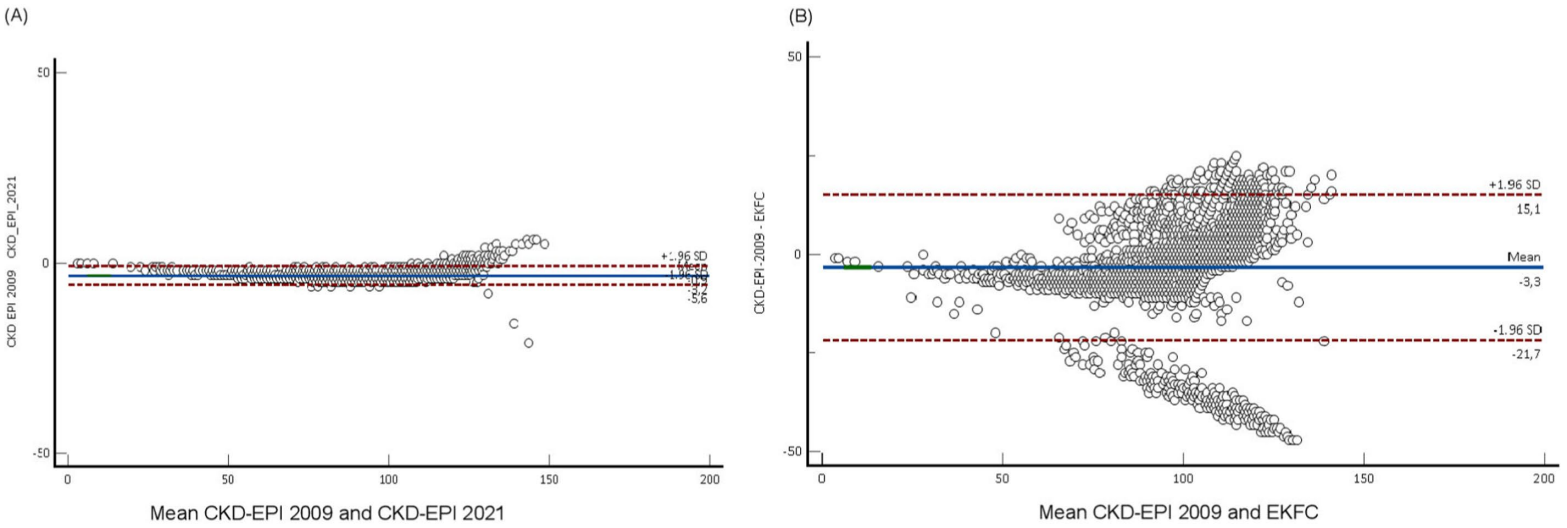
Bland Altman plots between eGFR CKDP-EPI 2021 and others (*n* = 9,557). **(A)** CKD-EPI 2021 formula, and **(B)** EKFC formula. The solid grey lines depict the mean difference, whilst the dashed lines reflect the standard deviation. A negative disparity signifies that each equation overestimates the GFR in comparison to the CKD-EPI 2009 equation.

The CKD-EPI 2009 equation exhibits a strong connection with all equations. The correlation values were 0.978 for CKD-EPI 2021 and 0.79 for EKFC (Figure 2). The EKFC and CKD EPI 2021 equations exhibited a decreased overall prevalence of reduced glomerular filtration rate (GFR) in comparison to the CKD-EPI 2009 equation. The mean eGFR change in the EKFC formula was considerably greater than the differences in the CKD-EPI 2021 when compared to the CKD-EPI 2009 (*p* < 0.001) (Figure 3).

### Different of CKD population between eGFR equations

The EKFC equation reported the lowest prevalence (1.03%) of reduced GFR (60 mL/min/1.73 m2), which corresponds to GFR categories G3a–G5. For all equations, the occurrence increased with age (Table 6). Furthermore, there was no disparity in the proportion of chronic kidney disease (CKD) between males and females.

**Table 6:** The occurrence of decreased eGFR below 60 mL/min/1.73 m2, as determined by three eGFR equations, categorised by age group and gender.

	Age (yr)	Male (*n* = 8,127) *n*,%	Female (*n* = 1,430) *n*,%	Total (*n* = 9,557) *n*,%
CKD-EPI 2021	17–19	0 (0)	0 (0)	0 (0)
	20–29	0 (0)	0 (0)	0 (0)
	30–39	4 (0,15)	1 (0,28)	5 (0,17)
	40–49	10 (0,35)	2 (0,45)	12 (0,36)
	50–59	13 (1,1)	1 (0,43)	14 (0,99)
	60–69	2 (6,9)	2 (5)	4 (5,8)
	70–79	0 (0)	1 (8,33)	1 (4,17)
	80–89	0 (0)	0 (0)	0 (0)
	Total	29 (0,36)	7 (0,49)	36 (0,38)
CKD-EPI 2009	17–19	0 (0)	0 (0)	0 (0)
	20–29	0 (0)	0 (0)	0 (0)
	30–39	4 (0,15)	1 (0,28)	5 (0,17)
	40–49	13 (0,45)	2 (0,45)	15 (0,45)
	50–59	16 (1,35)	1 (0,43)	17 (1,2)
	60–69	2 (6,9)	2 (5)	4 (5,8)
	70–79	0 (0)	1 (8,33)	1 (4,17)
	80–89	0 (0)	0 (0)	0 (0)
	Total	35 (0,43)	7 (0,49)	42 (0,44)
EKFC	17–19	0 (0)	0 (0)	0 (0)
	20–29	0 (0)	0 (0)	0 (0)
	30–39	2 (0,08)	1 (0,28)	3 (0,1)
	40–49	9 (0,31)	1 (0,23)	10 (0,3)
	50–59	10 (0,84)	0 (0)	10 (0,71)
	60–69	2 (6,9)	1 (2,5)	3 (4,35)
	70–79	0 (0)	0 (0)	0 (0)
	80–89	0 (0)	0 (0)	0 (0)
	Total	23 (0,28)	3 (0,21)	26 (0,27)

## Discussion

Creatinine will continue to be consistently utilized as kidney function marker in Indonesia in the future because of its low cost and availability. Consequently, eGFR estimations relying on serum creatinine levels will remain valuable. Even though the KDIGO has updated (CKD-EPI 2021) and collated consensus with the use of the eGFR formula, international expert interviews about the perspective of the usage of formulas in various parts of the world revealed a fundamental divergence. Europe, for example, recommends the new EKFC formula, whereas the US employs CKD-EPI 2021. The African continent may continue to use the CKD-EPI 2009 (18).

Many Asian countries have validated eGFR formulae based on their own characteristics. This is significant because the Asian population differs from the Caucasian population. Korea, Japan, and China, for example, have designed and modified the appropriate eGFR formula for each country’s population (%^9^–^12^, ^19^–^23^). Tae Dong and his colleagues determined that the EKFC formula was significantly more accurate in the Korean population than the CKD-EPI 2021 formula. Additionally, they observed that the CKD EPI 2009 formula exhibited a far greater level of precision than the CKD-EPI 2021 formula (19). By modifying the 2009 CKD-EPI formula, Matsou discovered that the Japanese population has its own formula (11). According to Xie, the CKD-Epicreat-Cys formula is the most appropriate formula for the CKD population with Diabetes Mellitus in China, whereas other formulas do not perform well (23).

We compared three creatinine-based eGFR formula in an adult Indonesian population. Currently, there was no data on those comparisons. The distribution of eGFR category exhibited significant variation among different equations, particularly for G1 and G2. Over 80% of the study group fell into the G1 category as determined by the EKFC equation. The cause of this variance could be connected to the research population’s eGFR distribution.

Our study demonstrated that the three formulas yield disparate estimations of renal function across different age groups. The CKD-EPI 2021 formula tended to overestimate renal function when compared to eGFRs calculated using the CKD-EPI 2009 and EKFC equations. The disparity in median ages among the participants is a particularly probable rationale for the study’s results. The variation in renal function determined by this three equations can be attributed to the inherent design of the estimations. During the ageing process, the amount of lean body mass decreases due to the combined effects of sarcopenia and the accumulation of fat tissue. Thus, Utsumi et al. (24) proposed the utilization of fat mass correction as an approach to enhance accuracy in the elderly population. Another study reveal that utilizing EKFC in laboratory settings for estimating eGFR provides a continuous assessment of GFR from early childhood to adulthood and old age, and might potentially serve as a substitute for CKD-EPI (25).

The mean CKD-EPI 2021 was 101.2 mL/min/1.73 m2 with a standard deviation of 16.2 mL/min/1.73 m2. As a result, there were several observations that deviated from the threshold value of 90 mL/min/1.73 m2 (%^26^–^28^). Our study showed the same result of lower eGFR in the elderly group in CKD-EPI 2009 compare to CKD-EPI 2021 and EKFC (27). This means that population-based studies are essential to determining the characteristics of the eGFR formula. In terms of race, Indonesia is made up of approximately 13,000 islands inhabited by almost 300 million people of different races (Proto-Malay in the west and Melanesia in the east). However, no research has been conducted to demonstrate the differences in creatinine levels between these two races. While race as a social construct is evolving, genetic and epidemiological differences remain relevant in medical research. It is well-documented that racial and ethnic differences influence creatinine metabolism, muscle mass, and consequently, eGFR estimations. Although Indonesia comprises multiple ethnic groups, no previous studies have specifically investigated differences in creatinine metabolism between these groups. Future research should explore whether ethnicity-based adjustments could enhance the accuracy of eGFR equations in the Indonesian population (29). However, it is more important to calculate median creatinine (30) in different region to maximize the use of non-race creatinine based equation to establish CKD since the differences in epidemiological variables, such as weight, height, muscle mass, age, and race, are known to have a significant impact on the creatinine-based model (30).

Our investigation revealed a significant discrepancy in creatinine levels between males and females. Generally, the Qcr levels in both male and female were lower compared to the European population. The difference in Qcr between male and female can be attributed to BMI in nearly all regions. It is well established that BMI, rather than the distribution of body fat, is considered an independent determinant in determining the rate at which creatinine is eliminated from the body in individuals who do not have diabetes. This suggests that lean mass, as opposed to fat mass, is a contributing factor in this correlation (31, 32). The variability in serum creatinine levels observed in the Indonesian population can be attributed to several factors, including differences in diet, muscle mass, and genetic background. Indonesia’s diverse dietary habits influence protein intake, which directly affects creatinine levels. Additionally, muscle mass, which varies between ethnic groups and geographical regions, plays a crucial role in creatinine production. Genetic variations affecting creatinine metabolism may also contribute to these differences. These factors must be considered when applying eGFR equations, as they can influence the accuracy of renal function estimates. Our findings suggest that while CKD-EPI 2021 tends to overestimate GFR, EKFC has the lowest prevalence of CKD classification, indicating that further validation with measured GFR methods is essential to determine the most appropriate equation for clinical use in Indonesia.

A cut-off GFR of 60 mL/min/1.73 m2 is a crucial criterion in assessing whether someone has CKD. This study revealed a low proportion of patients with CKD using the EKFC formula in comparison to the CKD-EPI 2009. This demonstrates that the application of eGFR is not straightforward and it has a wide impact on determining morbidity and long-term treatment finance. As a result, we propose that several eGFR formulas should be validated using measured GFR to determine which eGFR formula is closest to the Indonesian population. It is also necessary to confirm several clinical contexts, such as individuals with diabetes and hypertension, as contributors to the majority of CKD causes before they may be employed in patients.

This study demonstrates the correlation between each equation using CKD-EPI 2009 as the corresponding comparator. It indicates that there was a strong association between EKFC and the CKD-EPI 2009 equation. However, the existence of proportional bias indicates that the two equations do not demonstrate equal concordance throughout the entire range of eGFR values. As the values of eGFR rose, more noticeable differences were observed. Similar with this finding, study conducted by Lee et al. revealed that age is a more influential factor in predicting the disparity between the two eGFR equations. Furthermore, their data indicate that eGFR can vary significantly in the age group of 18–25 years (33). Furthermore, this provides evidence for implementing the suggested age limit of 26 years when calculating the median (Q) value in the EKFC equation, in conjunction with the previously established age limit of 40 years (7, 34).

The Bland–Altman analysis demonstrated a systematic bias in eGFR estimations, with CKD-EPI 2021 showing an upward deviation compared to CKD-EPI 2009 and EKFC. While Lin’s concordance correlation coefficients indicated a strong correlation between CKD-EPI 2009 and CKD-EPI 2021 (r = 0.978), the agreement with EKFC was lower (r = 0.79), suggesting greater discrepancies in classification. The reclassification rates observed between formulas highlight the potential clinical impact of choosing one equation over another, particularly in borderline cases of CKD diagnosis.

It is important to emphasize that insufficient data exists on the prevalent formula used by clinicians in Indonesia to evaluate renal function. Based on our experience in the laboratory, we report the CG, MDRD, and CKD-EPI because these formulas are the commonly requested. We did not involve CG and MDRD equations in this evaluation since those formula are outdated and full of limitations. In 2009, CKD-EPI collaboration suggested a new equation and then improving the equation in 2021 to exclude the racial aspect, which was deemed discriminatory (6). This equation was derived using data collected from various study cohorts, which included individuals who were in a healthy state. However, this equation has received considerable criticism from European perspectives, including the study’s conclusion, the fact that it performs insufficiently in non-Black populations (overestimating non-Black people) (29). The EKFC released a new development model in 2021 (7). It largely uses European data (19,629 cases), and also USA data (12,854 US cases). Plasma creatinine levels were measured using assays that are traceable to IDMS standards, and GFR was measured using a variety of established reference procedures (mostly iohexol plasma clearance). The EKFC equations can be utilized to calculate the GFR in the United States. This allows for the incorporation of self-reported race or unknown race, based on the patient’s preference as documented in hospital registration data (35). Among the three equations evaluated, CKD-EPI 2021 showed a higher estimated GFR compared to CKD-EPI 2009 and EKFC, suggesting a risk of overestimation in clinical practice. EKFC, on the other hand, exhibited a higher agreement with CKD-EPI 2009 in classifying reduced GFR (<60 mL/min/1.73m^2^), indicating its potential as a suitable equation for the Indonesian population. However, without measured GFR validation, it is difficult to ascertain which equation provides the most accurate estimation of kidney function in this population. Prospective studies incorporating direct GFR measurements are necessary to confirm these findings.

This study has the advantage of being conducted in several areas throughout Indonesia, including young to old adults, and it uses creatinine levels defined as IDMS. However, this study has limitations because we did not compare GFR using iohexol or inulin clearance as a reference technique. Another key consideration is the utilization of Cystatin C and/or a mixture of plasma creatinine in analyzing the eGFR that had showed more accuracy than using creatinine alone (6, 36). There is also a demographic skew of the study population, with 85% of participants being male and the majority coming from Java Island (which represents 70% of the population, i.e., 180 million people) (37). This distribution reflects the nature of the dataset, which was obtained from occupational health screenings where male employees predominate. While the large sample size provides meaningful insights, the findings may not be fully generalizable to the broader Indonesian population, particularly female individuals and those from underrepresented regions. There were a few case of CKD participants in this study, therefore another study with more data CKD participants will be required. Finally, this study has a retrospective design, which limits our ability to control for confounding factors such as comorbidities, dietary intake, and hydration status. Additionally, serum creatinine levels were obtained from routine medical examinations rather than a standardized clinical trial setting. While retrospective data provide valuable insights into real-world eGFR performance, future prospective studies should incorporate additional clinical parameters to enhance the robustness of findings.

Given the discrepancies observed in eGFR estimations across different formulas, future research should prioritize validation studies using measured GFR methods, such as iohexol or inulin clearance. Additionally, studies including participants with chronic kidney disease, diabetes, and hypertension would help determine which eGFR equation performs best in high-risk populations. Evaluating cystatin C-based equations as an alternative to creatinine-based formulas may also be beneficial, particularly for populations with variable muscle mass.

In conclusions, there were significant variations in eGFR across adult population in Indonesia regardless of the equation utilized. These discrepancies were observed not only in those with normal or slightly reduced eGFR, but also in those with CKD. Hence, it is imperative to conduct future studies in diverse clinical settings using mGFR in order to validate the most accurate equation for eGFR in Indonesia.

## Data Availability

The raw data supporting the conclusions of this article will be made available by the authors, without undue reservation.
